# Diagnostic performance of lung ultrasound for transient tachypnea of the newborn: A meta-analysis

**DOI:** 10.1371/journal.pone.0248827

**Published:** 2021-03-29

**Authors:** Lili He, Yinghua Sun, Wei Sheng, Qiong Yao

**Affiliations:** 1 Department of Ultrasound, Children’s Hospital of Fudan University, Shanghai, China; 2 Institute of Pediatrics, Children’s Hospital of Fudan University, Shanghai, China; 3 Shanghai Key Laboratory of Birth Defects, Shanghai, China; 4 Department of Radiology, Children’s Hospital of Fudan University, Shanghai, China; Zagazig University, EGYPT

## Abstract

Several recent studies demonstrated that lung ultrasound could achieve desired diagnostic accuracy for transient tachypnea of the neonate (TTN). However, the diagnostic performance of lung ultrasound for TTN has not been systematically studied to date. This meta-analysis aimed to investigate the performance of lung ultrasound in diagnosing TTN. The relevant literature was searched in PubMed, Medline, the Cochrane Library, and Embase databases without any restriction in terms of language and time until January 31, 2021. Studies that assessed the diagnostic performance of lung ultrasound for TTN were included. Seven studies with 1514 participants were summarized. The lung ultrasound provided more accurate performance for diagnosing TTN with pooled sensitivity and specificity of 0.67 [95% confidence interval (CI) = 0.63–0.71] and 0.97 (95% CI = 0.95–0.98), respectively. A higher summarized area under the summary receiver operating characteristic curve was observed as 0.9906. Lower sensitivity and area under the curve (AUC) of B-lines for TTN were observed as 0.330 (95% CI = 0.27–0.38) and 0.5000, respectively. Lung ultrasound provided highly accurate AUC, sensitivity, and specificity in detecting TTN. Large-scale studies are warranted in the future to confirm these results.

## Introduction

Transient tachypnea of the neonate (TTN), also designated as wet lung, was first defined as early as in 1966 and was the consequence of the clinical manifestation caused by the postponed clearance of fetal lung fluid [1]. TTN is one of the most frequent causes of respiratory distress in neonates and is generally transient. The symptoms of TTN overlap with the manifestations of a wide variety of pathophysiologic states and usually improve in 24 or 48 h. However, the respiratory disorder may sporadically become more severe. The diagnosis of neonatal TTN has always been a dilemma due to the lower sensitivity and specificity of clinical signs and symptoms [2].

Since 2007, lung ultrasound for the diagnosis of neonatal lung diseases, such as neonatal respiratory distress and TTN, has been emerging as a modality of diagnosis gradually accepted in recent years. Previous studies have shown the precise and stable performance of lung ultrasound in diagnosing neonatal lung pathology [3–6]. Chest x-ray and computed tomography (CT) have been widely used to diagnose lung pathology and identify the pathogenesis of TTN [7], but they lead to radiation exposure of neonates. Moreover, lung ultrasound is less expensive, easily trainable for operators, and faster for obtaining results. Although it cannot completely substitute the chest x-ray, its use may reduce the use of chest x-ray in clinical settings with clear benefits.

Recently, several studies demonstrated that lung ultrasound obtained a significantly improved diagnostic accuracy for TTN [5,8]. However, the diagnostic performance of lung ultrasound for TTN has not been studied systemically to date. Therefore, the present study was conducted to investigate the diagnostic performance of lung ultrasound for TTN.

## Material and methods

This study was performed according to the Handbook of Preferred Reporting Items for Systematic Reviews and Meta-analyses 2019 Statement [9].

### Literature search

PubMed, Medline, the Cochrane Library, and Embase were searched for the relevant literature without any restriction in terms of language and time until January 31, 2021. The joint and individual key terms such as “Ultrasound,” “Ultrasonic,” “Transient Tachypnea,” “Infant,” “Newborn,” “Infantile,” and “Neonatal” were used for searching the databases. To include more studies, all relevant references and books were explored. Google Scholar was used to search the literature that referenced relevant studies.

### Eligibility criteria

The eligibility criteria for the present meta-analysis were as follows: (1) infants that diagnosed with transient tachypnea; (2) detection was performed using lung ultrasound; (3) studies reported sufficient results on constructing 2 × 2 contingency tables; (5) studies were published in English; and (6) studies conducted on the same population with more detailed data or most recently published studies.

Case reports, letters, comments, reviews, studies conducted on animal models or experiments *in vitro*, studies not published in English, and studies for which data were not available were excluded from the analysis.

### Data extraction

All studies obtained from the aforementioned databases were evaluated independently by two authors. Any disagreements on their inclusion were discussed and resolved with the help of a third author. All the needed data were extracted using standardized tables. Any disagreements were discussed with a third reviewer until a consensus was reached. For each study meeting the eligibility criteria, the following data were extracted: characteristics of the studies (first author, published year, country, etc.), features of participants (age, nation, sex ratio, etc.), features of lung ultrasound, and true-negative (TN), false-negative (FN), true-positive (TP), and false-positive (FP) results for each study.

The right and left lungs were analyzed separately, and an independent diagnosis was made for each lung. A normal lung was defined as a thin, hyperechoic pleural line with normal pleural sliding, predominantly horizontal A-lines and possibly a few nonconfluent B-lines due to the substantial liquid content of the neonatal lung. TTN was defined as an interstitial syndrome with either diffuse noncompact B-lines or a gradient of echogenicity between inferior and superior areas corresponding to the double-lung point.

### Quality assessment

The quality assessment was independently conducted and crosschecked by two authors with the Quality Assessment of Diagnostic Accuracy Studies 2 (QUADAS-2) [10]. The quality assessment comprised four domains: (1) selection of participants; (2) index test, which described the conduction and interpretation of the results; (3) reference standard, which described the conduction of reference standard and interpretation of the results; and (4) flow of the participant inclusion and exclusion.

### Statistical analysis

The sensitivity and specificity of lung ultrasound for each study was assessed using TN, FN, TP, and FP. The pooled sensitivity, pooled specificity, and respective 95% confidence intervals (CIs) across all included studies were summarized using the random-effects models. The standard error (SE) and 95% CI, summary receiver operating characteristic curve (SROC), and area under the curve (AUC) for each study were also calculated. The DerSimonian–Laird random-effects models were employed to assess the AUC of the SROC curves due to heterogeneity. The 95% CIs of the pooled metric were compared to assess the relative performance of the technique. The *I*^2^ statistics were calculated to evaluate the consistency of the effect sizes. The heterogeneity for each study was categorized as low, moderate, and high according to the values of *I*^2^ being lower than 25%, between 25% and 50%, and higher than 75%, respectively [11]. Begg’s rank correlation [12] and Egger’s weighted regression methods [13] were used to assess the publication bias. Review Manager (version 5.3, The Cochrane Collaboration, Oxford, UK) and Stata 15.0 (Stata Corporation, TX, USA) were used for data analyses. Stata 15.0 was also employed to calculate the result for Begg’s and Egger’s tests. *P* values less than 0.05 indicated statistically significant differences.

## Results

### Study selection

**Fig 1:**
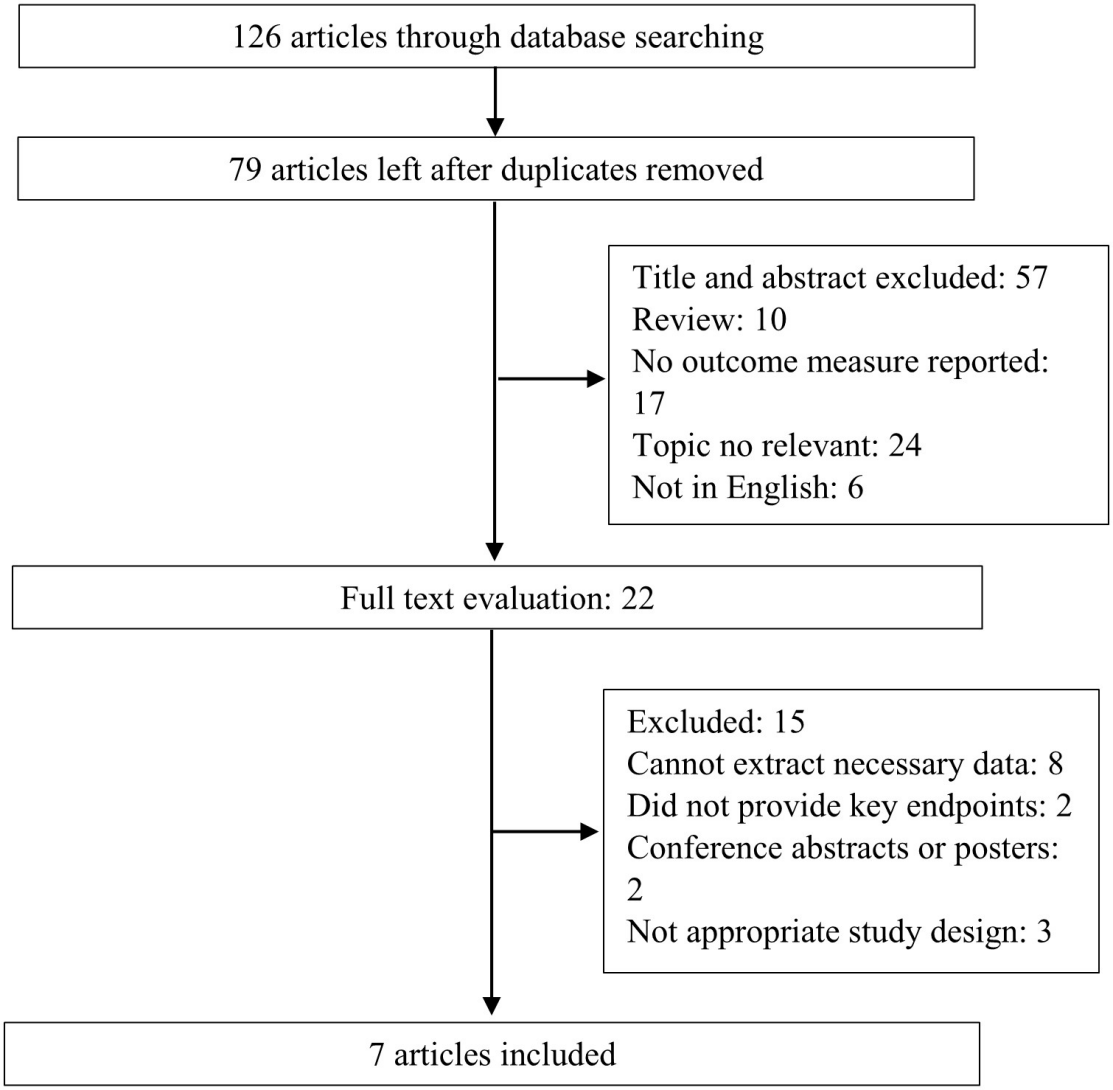
Flow chart of the study selection process.

The search strategy yielded 126 studies as potentially relevant studies; 47 studies were excluded due to duplication. Of the remaining 79 studies, 57 were excluded after browsing the titles or abstracts. Ultimately, seven studies [5,8,14–18] were included for data extraction and analysis. The flow chart for study selection is presented in Fig 1.

### Study characteristics

Seven studies (1514 participants) were included in the present meta-analysis. Table 1 shows the characteristics of the study participants; the sample size ranged from 26 to 1013. The studies were published from 2007 to 2019. Two of the studies were performed in China [15,16], two in Italy [8,14], one in India [17], one in Egypt [5], and one in France [18].

**Table 1:** Characteristics of study participants.

Studies included	Country	Study design	Gestational age (week) ^a^	Time between CXR and LUS	Diagnostic method	LUS operator	LUS equipment	Transducer frequency	Control
Copetti et al., 2007 [14]	Italy	Prospective	34.2 ± 1.01	<24 h	CXR	Pediatrician and cardiologist	Megas CVX Esaote Medical Systems	10 MHz	Healthy newborn infants
Liu et al., 2014 [15]	China	Prospective	27 ± 3 to 36 ± 2/28 ± 3 to 36 ± 1 ^b^^,^ ^c^	<24 h	CXR	Radiologist	GE Medical Systems	9–12 MHz	Without lung disease
Vergine et al., 2014 [8]	Italy	Prospective	34.5 ± 2.6/30.3 ± 3.7 ^c^	<24 h	Clinical Diagnosis and CXR	Radiologist	GE Medical Systems	10–12 MHz	Respiratory distress syndrome
Liu et al., 2016 [16]	China	Prospective	25 ± 4 to 41 ± 3 ^c^	<24 h	CXR	Radiologist	GE Voluson E6, E8 and Logiq C9 ultrasound	10–12 MHz	Other cases
Rachuri et al., 2017 [17]	India	Prospective	34.5 ± 3.2/35.9 ± 2.7	<24 h	CXR	Radiologist	Philips machine	10–12 MHz	Nonrespiratory illness
Ibrahim et al., 2018 [5]	Egypt	Prospective	37.3 ± 1.7/38.2 ± 1.6	<24 h	CXR	Expert	Philips HD7	7–12 MHz	Nonrespiratory illness
Grimaldi et al., 2019 [19]	France	Prospective	25–41 ^c^	<24 h	CXR	Neonatologists	Philips HD100 device	5–12 MHz	No lung disease

Abbreviation: CXR, Chest x-ray; GE, General Electric Company; HD, high definition; LUS, lung ultrasound; MHz, mega Hertz.

^a^Gestational age (week) ± standard deviation.

^b^Control gestational age.

^c^Range of gestational age.

### Study quality assessment

**Fig 2:**
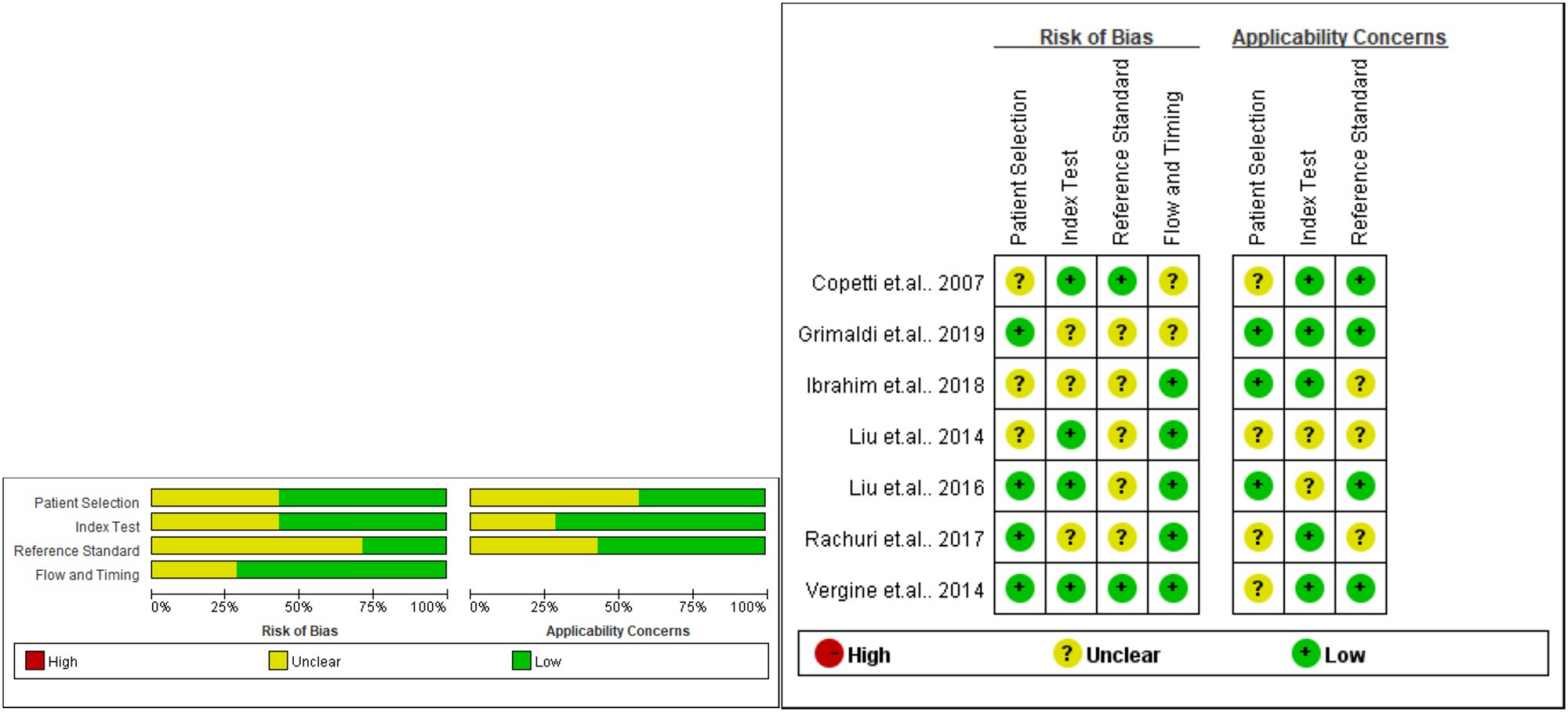
Risk-of-bias assessment of included studies.

Two reviewers independently evaluated each included study in the present meta-analysis according to the guideline for QUADAS-2. For all the items of QUADAS-2, almost each domain had unclear risks due to missing information or non-blinded selection of grouping. None of the domains was judged as high risk. The results for quality assessment using QUADAS-2 are presented in Fig 2.

### Observation indexes

The majority of the studies reported the observation indexes for lung ultrasound, including pleural-, A-, and B-lines, as well as the double-lung point (DLP). Five studies reported the diagnostic performance of DLP for the TTN [8,14,15,17,18], and two reported the results on B-lines [5,16].

### Diagnostic accuracy of DLP for TTN

**Fig 3:**
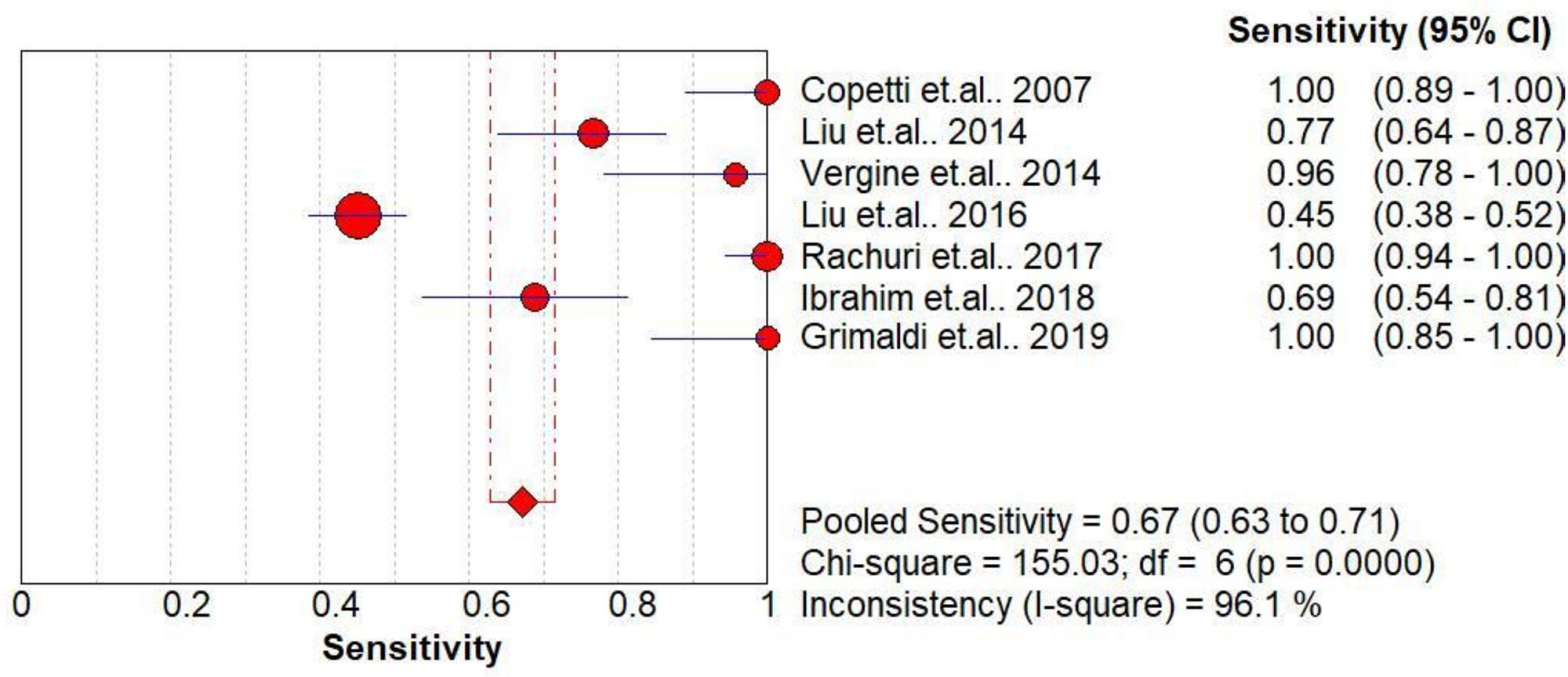
Summary sensitivity analysis of the double-lung point for transient tachypnea of the neonate.

**Fig 4:**
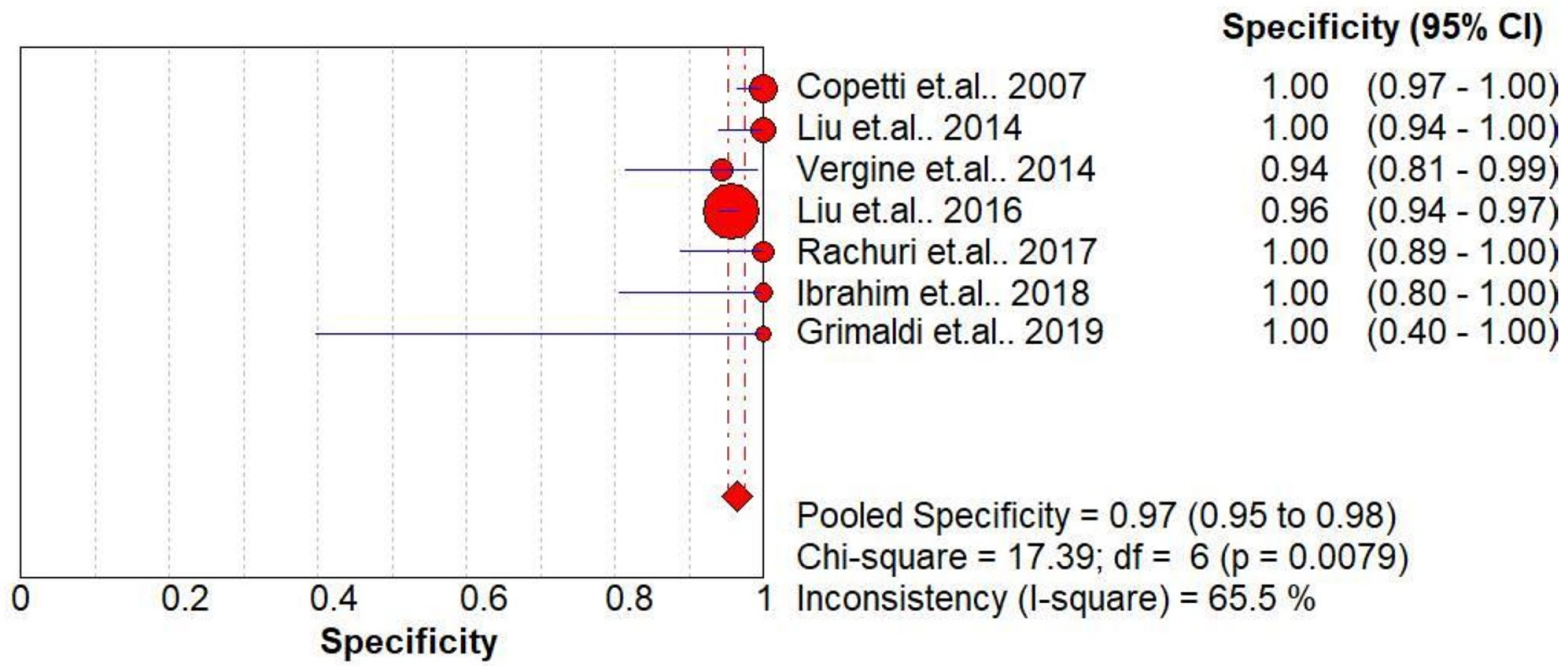
Summary specificity analysis of the double-lung point for transient tachypnea of the neonate.

**Fig 5:**
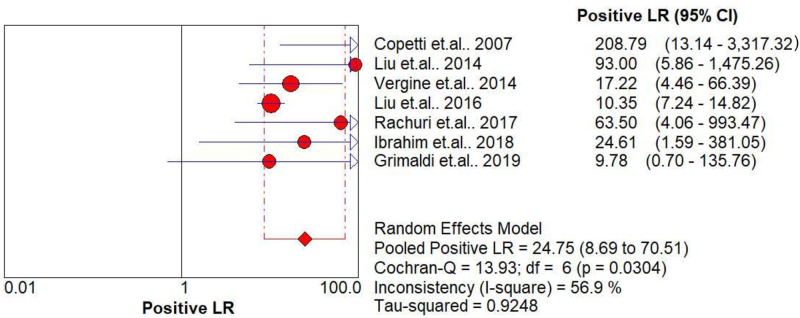
Summary of positive likelihood ratio of the double-lung point for transient tachypnea of the neonate.

**Fig 6:**
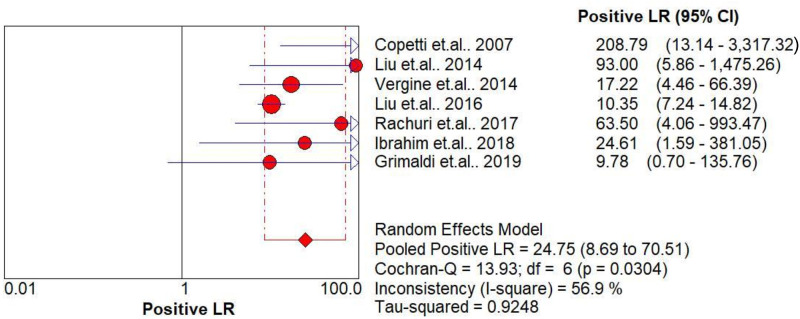
Summary of negative likelihood ratio of the double-lung point for transient tachypnea of the neonate.

**Fig 7:**
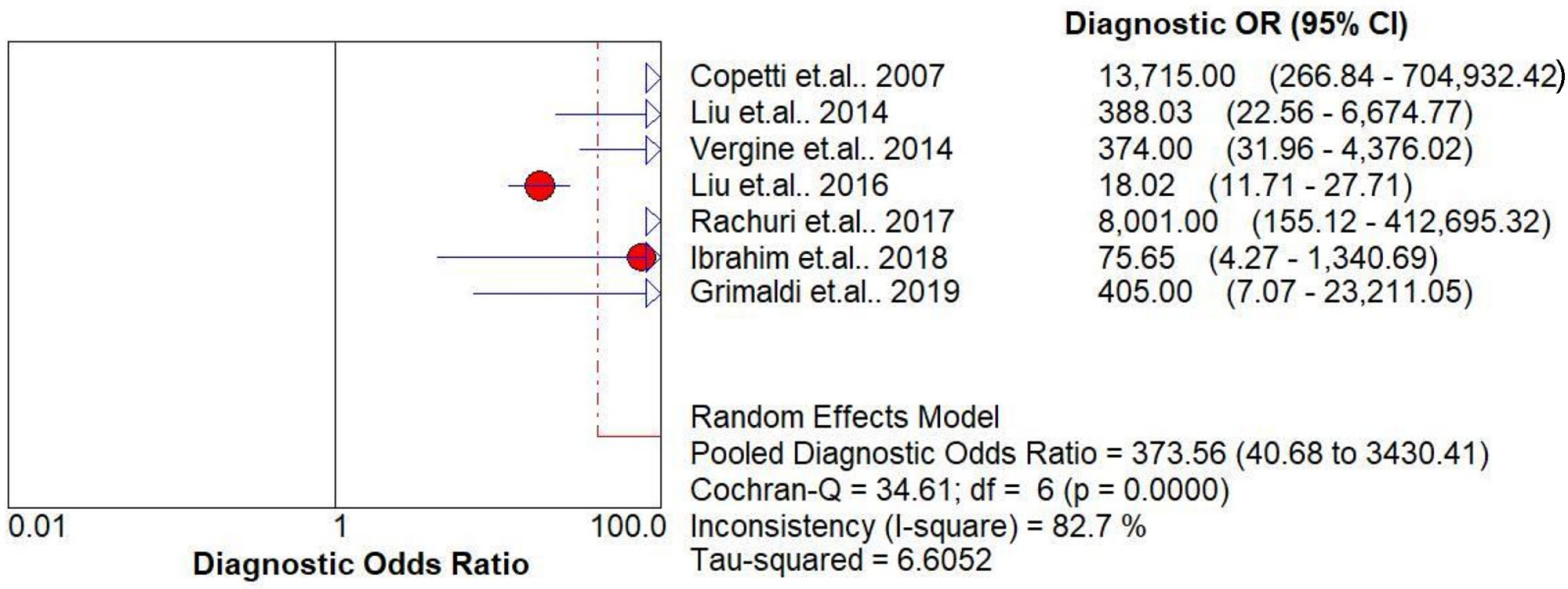
Summary of diagnostic odd’s ratios of the double-lung point for transient tachypnea of the neonate.

**Fig 8:**
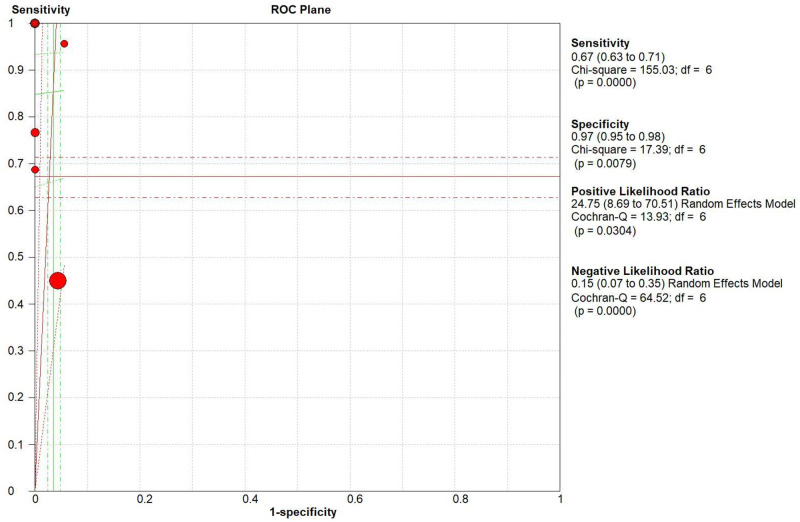
Summary of the pooled ROC curve of the double-lung point for transient tachypnea of the neonate.

**Fig 9:**
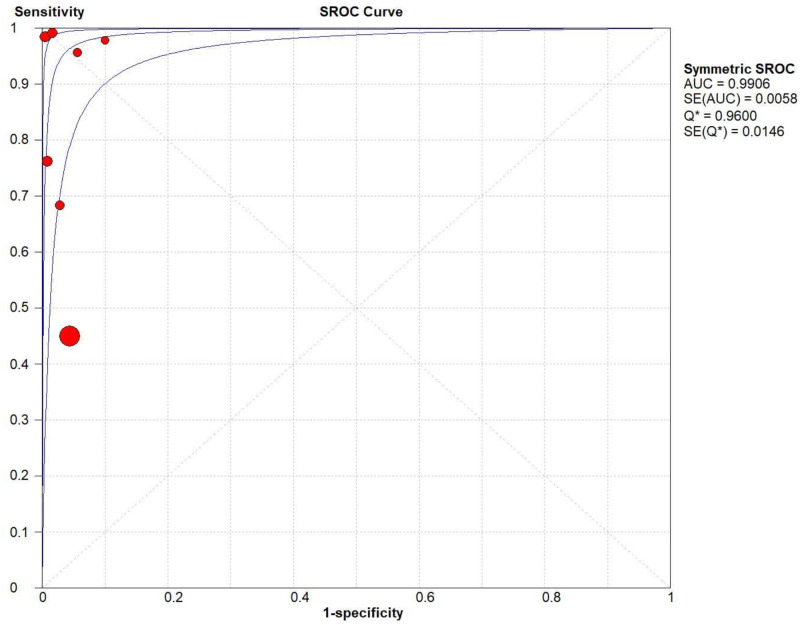
Summary of the pooled area under the SROC curve of the double-lung point for transient tachypnea of the neonate.

The pooled diagnostic accuracies of DLP in diagnosing TTN are shown in Figs 3–8. The DLP showed moderate sensitivity as 0.67 (95% CI = 0.63–0.71) and better specificity as 0.97 (95% CI = 0.95–0.98). The pooled area under the SROC curve was 0.9906 with an SE of 0.0058 (Fig 9). The forest plots suggested that the heterogeneity was high with almost all the *I*^*2*^ values exceeding 75%.

### Subgroup analysis

The pooled results for the studies grouped by study design and country are shown in S1–S6 Figs. The studies from Asian countries (S1 Fig) had similar sensitivity (0.67, 95% CI = 0.63–0.71) compared with the studies conducted in Europe (0.65, 95% CI = 0.60–0.70, S7 Fig).

### Diagnostic accuracy of B-lines for TTN

Two studies reported the data on the diagnostic accuracy of B-lines for TTN. The results are presented in S5 and S6 Figs. The diagnostic accuracy of B-lines for TTN was observed with a lower sensitivity of 0.330 (95% CI = 0.27–0.38), and the AUC was 0.5000.

### Meta-regression and subgroup analyses

Several covariates were included for the meta-regression: study design (case–control versus prospective studies), location of the studies (Asia versus Europe), and operator for lung ultrasound (radiologists versus others). The meta-regression analysis demonstrated that the operator might explain the heterogeneity observed (Table 2).

**Table 2:** Meta-regression by inverse variance weights.

	Coefficient	Stand error	*P* value	RDOR (95%CI)
**All variables**				
Cte	3.502	3.1713	0.3501	NA
S	0.530	0.2486	0.1225	NA
Country	–0.820	1.0008	0.4727	0.44 (0.02–10.65)
Operator	2.671	1.1193	0.0970	14.46 (0.41–509.58)
**By operator**				
Cte	2.052	1.3221	0.1813	NA
S	0.623	0.1691	0.0143	NA
Country	NA	NA	NA	NA
Operator	2.954	1.0664	0.0394	19.18 (1.24–297.45)

Abbreviations: CI, confidence interval; Cte, constant coefficient; NA, not available; RDOR, relative diagnostic odds ratios; S, S coefficient.

### Publication bias

No publication bias was observed (all *P* values were >0.05) according to Begg’s rank correlation analysis and Egger’s weighted regression tests (S1 Table).

## Discussion

This meta-analysis was novel in evaluating the diagnostic value of combined lung ultrasound for detecting TTN. Seven studies reported the diagnostic performance of DLP for TTN, and two reported the results on B-lines. The pooled results from seven studies demonstrated that lung ultrasound provided accurate performance for diagnosing TTN by DLP with summarized sensitivity and specificity as 0.67 (95% CI = 0.63–0.71) and 0.97 (95% CI = 0.95–0.98), respectively. The pooled SROC was 0.9800.

Breathing difficulty was one of the most common neonatal critical diseases that needed immediate therapy. TTN was one of the most common reasons for breathing difficulty and could lead to severe neonatal dyspnea. Previous studies showed that TTN accounted for approximately 33%–50% of neonatal breathing difficulty in patients [20,21]. The accurate diagnostic method for TTN would be the crucial element to improve the prognosis of patients. In previous studies [1,2], lung ultrasound showed high sensitivity and specificity in diagnosing TTN and was effective in investigating the main neonatal respiratory diseases. In this study, lung ultrasound was demonstrated to be a practical tool for diagnosing TTN. Some patients had a normal chest x-ray (CXR) despite having the clinical symptoms of TTN. Chest CXR and CT scan have been widely used to find the cause of respiratory distress in neonates. CXR and CT were broadly used to eliminate differential diagnoses or complications of underlying diseases. However, CXR and CT led to radiation exposure of neonates, making it difficult for the neonatologist. The avoidance of ionizing radiation could be done through prudent clinical use. Lung ultrasound was a less ideal tool for examining the normal aerated lungs because ultrasound could not travel smoothly through the air. Lung ultrasound is highly operator dependent; an inherent source of potential error may exist, although using a standard approach helps limit operator dependency. The evaluation of the lung became possible due to advances in ultrasound technology in the last few years. Moreover, the international evidence-based recommendations standardized the main indications of lung ultrasound [22] and also verified ultrasound superiority over CXR in diagnosing TTN [15]. In the clinical settings, the usefulness and the possible impact of lung ultrasound in daily practice might tremendously benefit newborns.

The possible etiology for TTN may be the deferred elimination for pulmonary fluid caused by the immaturity or postpone of the pulmonary epithelium transformed from chloride secretion to enable sodium chloride reabsorption. The respiratory symptoms of TTN are usually transient and relatively mild, and the treatment is usually oxygen supplementation alone. DLP is the demarcation point formed between the upper and lower lungs on an ultrasound due to the differences in the severity or nature of lesions. In the present study, DLP was presented in a majority of newborns with TTN. In the past, the appearance of DLP was deemed a specific and sensitive sign for TTN. In the present study, DLP was found to have higher accuracy for TTN. DLP, exposing a higher water content at the bases compared with the apical regions, may be interpreted by recalling the dynamics of lung water drainage in hours after delivery.

The present study had some limitations. First, only seven studies were included, and most of the studies had limited sample size. The small sample size might have reduced the credibility and stability of the results. Second, the heterogeneity in the analysis remained high due to the involvement of different types of operators for lung ultrasound in the studies. The differences in operators in studies might have led to heterogeneity and reduction in the stability of the results. Third, various ultrasound devices were used to assess TTN. The different parameters of ultrasound equipment might have also caused the heterogeneity. However, it might not be possible to ensure that all parameters were kept constant. Fourth, language bias might also exist because all included studies were published in English only.

## Conclusions

In conclusion, lung ultrasound had highly accurate AUC, sensitivity, and specificity in detecting TTN. Nevertheless, the conclusions were based on only seven studies, and hence the heterogeneity needs to be taken into account. More studies should be conducted in the future to further assess and compare the use of lung ultrasound for detecting TTN.

## Supporting information

S1 Checklist PRISMA 2009 checklist. (DOC)

S1 Fig Subgroup analysis of studies from Asian countries using the pooled ROC curve of the double-lung point for transient tachypnea of the neonate. (DOC)

S2 Fig Subgroup analysis of studies from Asian countries using the pooled area under the SROC curve of the double-lung point for transient tachypnea of the neonate. (DOC)

S3 Fig Subgroup analysis of studies conducted in Europe using the pooled ROC curve of the double-lung point for transient tachypnea of the neonate. (DOC)

S4 Fig Subgroup analysis of studies conducted in Europe using the pooled area under the SROC curve of the double-lung point for transient tachypnea of the neonate. (DOC)

S5 Fig Summary of the pooled ROC curve of B-lines for transient tachypnea of the neonate. (DOC)

S6 Fig Summary of the pooled area under the SROC curve of B-lines for transient tachypnea of the neonate. (DOC)

S7 Fig Subgroup analysis of studies conducted in Europe of the pooled ROC curve of double-lung point for transient tachypnea of the neonate. (DOC)

S1 Table Publication bias of summarized outcomes. (DOC)

S1 File PRISMA 2009 flow diagram. (DOC)
