# Supplementary material for: Diagnostic performance of lung ultrasound for transient tachypnea of the newborn: A meta-analysis
Source: PLoS One. 2021 Mar 29;16(3):e0248827. doi: 10.1371/journal.pone.0248827 (PMC8006999; doi:10.1371/journal.pone.0248827)
Supplement: S4 Fig — (DOC) [file pone.0248827.s005.doc]

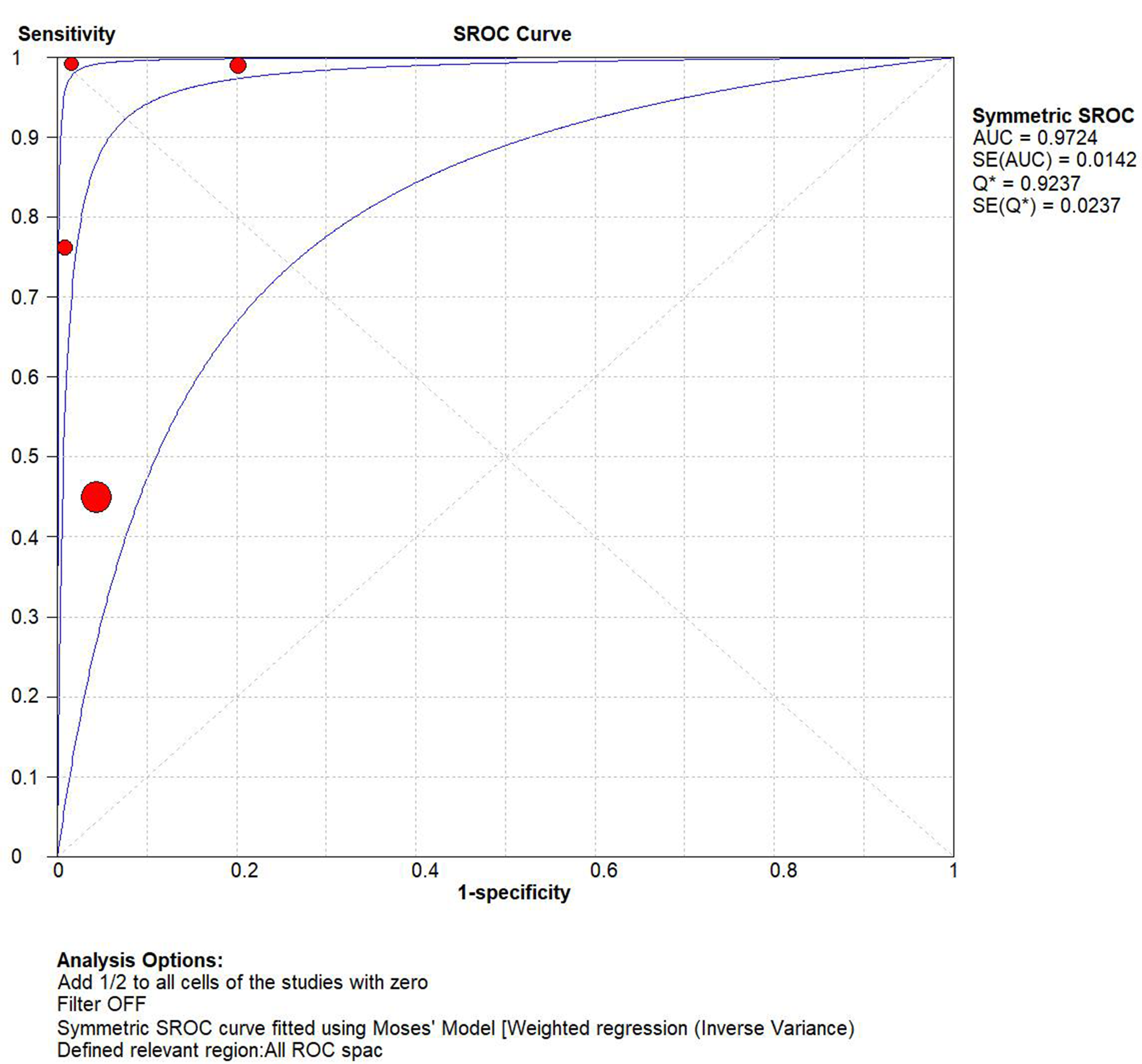


Supplementary Figure 4. Subgroup analysis of studies conducted in Europe using the pooled area under the SROC curve of the double-lung point for transient tachypnea of the neonate.
