# Supplementary material for: Diagnostic performance of lung ultrasound for transient tachypnea of the newborn: A meta-analysis
Source: PLoS One. 2021 Mar 29;16(3):e0248827. doi: 10.1371/journal.pone.0248827 (PMC8006999; doi:10.1371/journal.pone.0248827)
Supplement: S6 Fig — (DOC) [file pone.0248827.s007.doc]

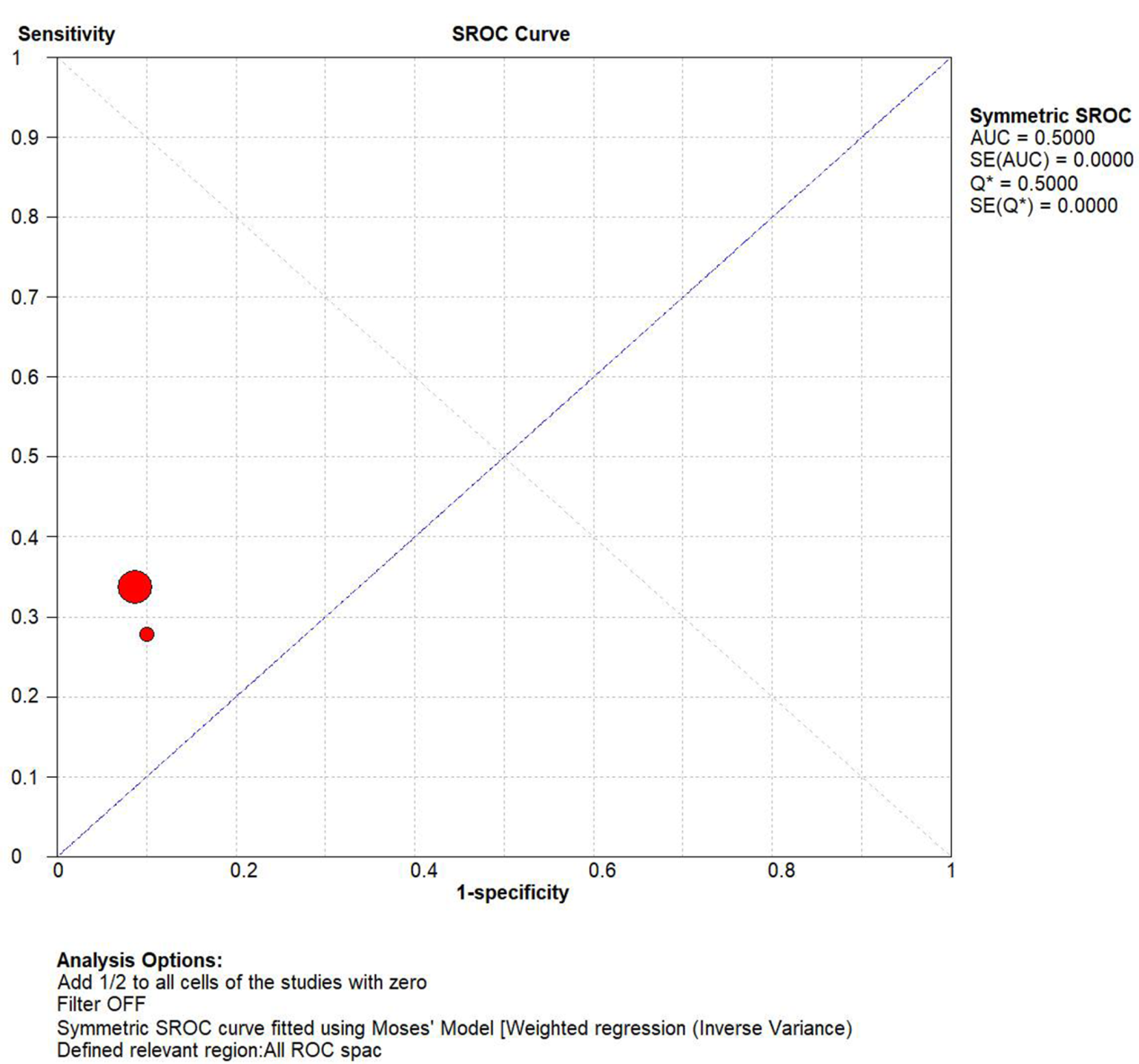


Supplementary Figure 6. Summary of the pooled area under the SROC curve of B-lines for transient tachypnea of the neonate.
