# Supplementary material for: Diagnostic performance of lung ultrasound for transient tachypnea of the newborn: A meta-analysis
Source: PLoS One. 2021 Mar 29;16(3):e0248827. doi: 10.1371/journal.pone.0248827 (PMC8006999; doi:10.1371/journal.pone.0248827)
Supplement: S1 Table — (DOC) [file pone.0248827.s009.doc]

**Supplementary materials**

**Table S**1. Publication bias of summarized outcomes

| **Outcomes** | **Begg (*P* value)** | **Egger (*P* value)** |
| --- | --- | --- |
| Summarized sensitivity | 0.40 | 0.21 |
| Summarized specificity | 0.77 | 0.79 |
| Summarized positive likelihood ratio | 0.44 | 0.26 |
| Summarized negative likelihood ratio | 0.53 | 0.44 |
| Summarized diagnostic odds ratios | 0.78 | 0.45 |
| Summarized pooled area under the SROC | 0.45 | 0.21 |

SROC, Summary receiver operating characteristic curve.
