# Supplementary material for: Diagnostic performance of lung ultrasound for transient tachypnea of the newborn: A meta-analysis
Source: PLoS One. 2021 Mar 29;16(3):e0248827. doi: 10.1371/journal.pone.0248827 (PMC8006999; doi:10.1371/journal.pone.0248827)
Supplement: S1 File — (DOC) [file pone.0248827.s010.doc]

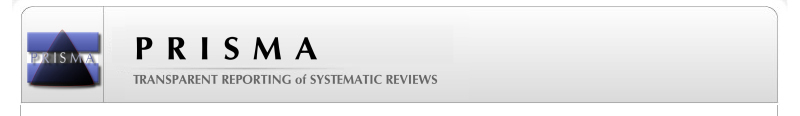
**PRISMA 2009 Flow Diagram**

**Screening**

**Inclusion**

**Eligibility**

**Identification**

Records identified through database searching
(*n* = 126)

Additional records identified through other sources
(*n* = 47)

Records after duplicates were removed
(*n* = 79)

Records screened
(*n* = 125)

Records excluded
(*n* = 103)

Full-text articles assessed for eligibility
(*n* = 22)

Full-text articles excluded, with reasons
(*n* =14)

Studies included in qualitative synthesis
(*n* = 0)

Studies included in quantitative synthesis (meta-analysis)
(*n* = 8)
